# MicroRNA Expression Relating to Dietary-Induced Liver Steatosis and NASH

**DOI:** 10.3390/jcm4111938

**Published:** 2015-11-16

**Authors:** Aida Zarfeshani, Sherry Ngo, Allan M. Sheppard

**Affiliations:** Developmental Epigenetics Group, Liggins Institute, The University of Auckland, 85 Park Road, Grafton, Auckland 1023, New Zealand; E-Mails: a.zarfeshani@auckland.ac.nz (A.Z.); s.ngo@auckland.ac.nz (S.N.)

**Keywords:** diet, miRNA, NAFLD, NASH

## Abstract

Health issues associated with excessive caloric intake and sedentary lifestyle are driving a modern “epidemic” of liver disease. Initially presenting in the clinic as an excessive accumulation of fat within hepatocyte cells (steatosis), the progression to more severe non-alcoholic steatohepatitis (NASH) in which liver damage and inflammation are overt features, is becoming increasingly common. Often developing as a sequela of obesity, non-alcoholic fatty liver disease (NAFLD) arises in almost one-third of people initially carrying excess hepatic fat and is likely the result of the liver’s limited capacity to cope with the modern-day levels of dietary fatty acids circulating in the blood. While routine imaging can readily assess the presence and level of “extra-hepatic fat”, a proper diagnosis of disease progression to NASH is currently only possible by liver biopsy. A general reluctance to undergo such screening means that the prevalence of NASH is likely to be under reported and, thus, risk assessment for future metabolic syndrome (MetS) markedly compromised. The seemingly inevitable progression to overt insulin resistance that characterizes MetS may in part be the consequence of the body’s attempt to cope with NAFLD by driving systemic insulin sensitivity and, thus, fatty acid breakdown. The potential significance of miRNAs in both physiological homeostasis and pathogenesis is increasingly appreciated and in the liver may contribute specifically to the regulation of lipid pathways and NAFLD progression. As such, they may have utility as molecular indicators for the accurate profiling of both initial risk and disease progression from simple steatosis to NASH, and further to fibrosis/cirrhosis.

## 1. Diet-Induced Fatty Liver Disease

**Figure 1:**
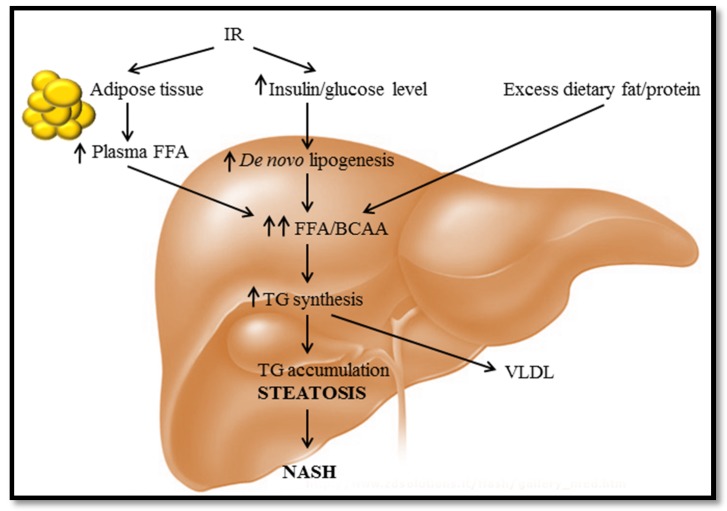
Hepatic triglyceride (TG) accumulation. FFAs are derived from the diet or synthesized *de novo* by hepatocytes. High levels of dietary protein intake (leading to increased circulating BCAA) can induce peripheral IR by inhibiting insulin signaling, resulting in increased uptake of glucose and TG storage in hepatocytes. Meanwhile, IR in adipose tissue reduces the inhibitory effect of insulin on lipoprotein lipase, resulting in increased lipolysis (*i.e.*, breakdown of TG) and, thus, to increased circulating FAs to be taken up by the liver. Hyperinsulinemia can also induce *de novo* synthesis of FFAs in hepatocytes, leading to hepatic TG synthesis. These pathways drive hepatic TG accumulation and ultimately may contribute to the development of NASH. IR: insulin resistance; FFA: free fatty acid; BCAA: branched-chain amino acid; NASH: non-alcoholic steatohepatitis; VLDL: very low-density lipoprotein.

A combination of dietary-based caloric excess and a sedentary lifestyle (confounded by socioeconomic factors) has led to population-wide weight gain and, subsequently, an increasing incidence of obesity-related comorbidities (e.g., non-alcoholic fatty liver (NAFLD), type 2 diabetes mellitus (T2DM) and cardiovascular diseases) [1,2]. In addition to driving the uptake and “storage” of excess circulating fatty acids (FAs) into peripheral tissues, this generalized metabolic syndrome (MetS) is also characterized by elevated triglyceride (TG) synthesis, leading to yet further fat accumulation, particularly in liver and adipose tissues [3] (Figure 1). The abnormal accumulation of fat in the liver (occurring in the absence of significant alcohol consumption) is a defining characteristic of NAFLD and begins with the intra-cytoplasmic accumulation of TG as liposomes around the hepatocyte nucleus. At more advanced stages, these vesicles increase in size, such that the nucleus is distorted and displaced to the periphery of the hepatocyte, a condition that is known as macrovesicular steatosis [4]. The pathological spectrum of NAFLD progresses then to non-alcoholic steatohepatitis (NASH), which is defined by the additional degeneration of the hepatocytes and sinusoidal fibrosis [4,5] and, finally, to end-stage cirrhosis, the main driver of liver transplant interventions [6,7].

Lipids are not only important as structural components of cell membranes (in the form of cholesterol and phospholipids) and energy storage (in the form of TG), but also serve as signalling molecules. Although generally sourced through the diet, fatty acids are also synthesized *de novo* as a normal function of hepatocyte cells [8]. However, abnormally elevated hepatic FA biosynthesis can precipitate glucose intolerance and insulin resistance (IR) as a consequence of the systemic attempt to restore homeostasis by promoting fat turnover [9]. By driving lipolysis, this adaptive mechanism unfortunately results in further increases in the circulating level of TG-derived free fatty acids (FFAs) [10]. Furthermore, the pathogenesis of IR is commonly accompanied by inflammation [11,12], which in turn stimulates the secretion of hepatokines [11,13,14,15]. Consequently, the progression of liver disease often occurs in parallel with that of MetS.

## 2. MicroRNAs in Metabolic Syndrome and NAFLD

MicroRNAs (miRNAs) are a class of endogenous, short, non-protein coding, single-stranded gene products, typically 20–22 nucleotides long [16]. The majority of miRNAs are intracellular [17] and encoded in the introns of protein-coding genes [18]. The primary miRNA is transcribed in the nucleus and subsequently integrated into the RNA-induced silencing complex in cytoplasm [19] to regulate the expression of target genes [20,21]. They are present in genomes across all eukaryotic organisms and are thought to modulate the expression of target genes post-transcriptionally via interactions with specific mRNAs [22].

MicroRNA activity is known to impact a diverse range of biological processes, including cellular growth and development, cardiovascular and cerebrovascular function, protein secretion and glucose and fatty acid metabolism [8,16,23,24,25]. Their potential importance for regulating metabolic homeostasis is also becoming clear. For example, the antisense targeting of just miR-122 in high-fat fed mice leads to a significant 30% decrease in circulating cholesterol levels [26,27], hepatic cholesterol and fatty acids biosynthesis and elevated fatty acid β-oxidation associated with a reduction in TG and hepatosteatosis [26]. Further, miR-33a and miR-33b are co-transcribed with their respective human host genes SREBP2 and SREBP1 [28], the sterol regulatory element-binding protein (SREBP) family of transcription factors being key regulators of many genes involved in FA and cholesterol biosynthesis, as well as TG and phospholipids production. miR-33a and miR-33b both target and act on the α-subunit of AMP-activated protein kinase (AMPK) [8,29,30]. In response to low cellular energy levels, AMPK decreases energy-consuming processes (such as FA biogenesis) to promote ATP synthesis. AMPK inhibits the activity of SREBPs and, by catalysing phosphorylation, activates their target substrates, such as acetyl-CoA carboxylase (ACC)-1. Therefore, inhibition of AMPK expression via miR-33 leads to the stimulation of SREBPs (and their target genes) to increase intracellular levels of TG, cholesterol and FAs. 

A number of miRNAs have been reported to be dysregulated in rodent models of NAFLD, obesity and T2DM, in some cases aligning with the changes observed in obese human patients with NAFLD and NASH [31,32,33]. Notably, miR-200a/b and miR-429, key members of the miR-200 family (miR-200a, miR-200b, miR-200c, miR-141 and miR-429), are all upregulated with disease. In addition, miR-451, miR-27a and miR-122 are downregulated in the liver of rats fed with a diet of high fat or high fructose or their combination [34]. Also reported as suppressed are miR-29c in diet-induced NASH [35] and miR-21, miR-29c and miR-451 in livers of *ob*/*ob* mice with fatty liver [36]. miR-34a is yet another species that is particularly associated with hepatic metabolic diseases, being highly expressed in patients with steatosis and NASH [32], as well as T2DM subjects [33]. In particular, miR-34a expression increases with the severity of NASH [37]. Castro *et al.* have demonstrated that miR-34a expression, upon stimulation by ursodeoxycholic acid (UDCA) (a potent inhibitor of apoptosis), inhibits p53 via the miR34a/sirtuin (SIRT)-1/p53 pathway in primary hepatocytes. In turn, p53 modulates miR-34a expression through a positive feedback loop and highlights the important role of miR-34a in regulating hepatocyte apoptosis and NAFLD progression [37]. The link with SIRT-1 is also clear evidence that miRNAs influence metabolism. SIRT-1 has recently been identified as a critical central metabolic regulator that is responsive to intracellular NAD^+^ levels and, by de-acetylating both histone and non-histone targets, alters the expression of genes involved in lipid and cholesterol synthesis and energy homeostasis, including proliferator-activated receptors (PPARs), proliferator-activated receptor gamma coactivator 1-α (PGC-1α), forkhead box-protein 1 (FOXO1), p53 and SREBPs [38,39,40]. 

MiR-335 is also highly expressed in obese mice [41] and is encoded within an intronic region of the imprinted gene MEST [42], although the transcriptional control of miR-335 is independent of the host gene [41]. While MEST has been reported to enhance the capacity for lipid storage in adipocytes [43], miR-335 is upregulated in the liver and adipose tissue of diabetic *db*/*db* mice and promotes lipid accumulation during adipocyte differentiation in the murine 3T3-L1 cell model [41]. It has also been linked with adipose tissue inflammation [44] and more recently shown to inhibit hepatic stellate cell activation and migration, suggesting a potentially positive role in reducing hepatic fibrosis [45]. Given that liver fibrosis and increased TG (both in liver and in circulation) are characteristics of NAFLD, miR-335 presents a potentially valuable therapeutic candidate for the diagnosis and treatment of NAFLD. In our own studies, we have found that circulating miR-335 is significantly decreased after weight loss in obese patients with T2DM who underwent gastric bypass (GBP) bariatric surgery and that this reduction in miR-335 level was strongly correlated with BMI [46]. It has previously been reported that circulating levels of the hepatokine fetuin A (FetA), a glycoprotein shown to promote lipid-induced insulin resistance [47], decrease after GBP surgery in obese subjects with T2DM [48]. Using the approach of RNA interference-mediated knockdown in the liver cell line HepG2, we have demonstrated that miR-335 mediates the increase in FetA expression that results from incubation with the FFA palmitate.

In these same studies, we also showed that the palmitate-induced increase in FetA expression is myostatin (MSTN)-dependent [46]. A member of the transforming growth factor-β (TGF-β) superfamily, MSTN is classically known as a regulator of skeletal muscle growth. However, elevated MSTN expression (circulating and intracellular) is associated with metabolic disorders, such as obesity [49] and type 1 diabetes mellitus (T1DM) [50]. The secreted form of MSTN is strongly correlated with human obesity [49] and weight loss [51], suggesting an emergent and important role for MSTN in the regulation of energy metabolism. Further, murine models of loss-of-function MSTN mutations [52], gene knockout [53] or pro-peptide overexpression [54] are all resistant to high fat diet-induced IR and obesity. Indeed, MSTN depletion attenuates adipose formation and reduces hepatic steatosis in high fat diet-induced obese mice [53]. In the context of NAFLD, the importance of miRNA in regulating MSTN function remains to be fully elucidated. However, we have shown that miRNA-dependent MSTN activity mediates both hepatic TG levels and biosynthesis in response to leucine, a branched-chain amino acid (BCAA), which is known to be elevated in obese and IR patients [55] and in subjects progressing from steatosis to NASH [56]. Notably, miR-143 and miR-92b modulated the MSTN-dependent regulation of key metabolic genes involved in glucose uptake and TG accumulation in HepG2 cells supplemented with leucine [55]. Given that elevated TG and insulin resistance are common features of NAFLD, miR-143 and miR-92b present potential candidate interventions in NAFLD, although how these miRNAs may be involved in the regulation of lipid homeostasis remains to be fully investigated. 

## 3. The Clinical Relevance of Exosomal Signalling in NAFLD

While an initial diagnosis of NAFLD can be made with modern imaging technologies, a definitive diagnosis of NASH requires confirmation by liver biopsy [57]. However, the invasive nature of this procedure often deters patient uptake, suggesting that the prevalence of NAFLD worldwide is likely to be understated. Furthermore, the inherent sampling variability associated with the biopsy process makes accurate histopathological diagnosis difficult and unreliable [57]. Meanwhile, the metabolites currently used to assess metabolic disease (such as ALT and AST) by computed tomography (CT) are not sufficiently correlated to liver disease to be specifically useful for accurate monitoring of progression, nor to account for differences between patients in various stages of NAFLD [58].

Early indications suggest that miRNA profiles measured in readily-sourced plasma and serum samples may however represent a new diagnostic approach for liver diseases [59]. Several differentially-expressed miRNA species have specifically been reported in plasma samples of NAFLD subjects. In particular, miR-16 is significantly overexpressed in both human and rats with steatosis/NASH compared to healthy subjects [32], while in patients with NAFLD, miR-21, miR-122, miR-192, miR-375, miR-19a, miR-19b and miR-146b are all significantly upregulated [32,59,60,61,62,63]. Furthermore, the level of expression for particular miRNAs appears to correlate with the severity of clinical histopathology, notably miR-181a, miR-34a, miR-122, miR-200 and miR-192, while miR-34a exhibits the strongest correlation with a histopathology score in mice with fat-induced liver injury [62]. Interestingly, increased miR-192, miR-375 and miR-122 distinguish more advanced NASH compared to simple steatosis, while miR-122 distinguishes liver fibrosis specifically [59]. 

In this review, we have described a number of miRNAs (summarized in Table 1) that are clearly associated with steatosis/NASH and have sought to highlight those that may underpin diagnostic profiles in “soluble biopsy” samples. Since miRNAs generally show a great degree of stability in extracellular environments (including blood) that contain active ribonucleases, it is clear that secreted miRNAs must be packaged to protect against degradation [17]. Generally, this involves incorporation into either exosomes [64] or RNA-binding protein complexes [65], and this enables their efficient recovery from biofluid clinical samples [66]. Notably, upregulation of both miR-122 and miR-155 following inflammation, and the related pathology alcoholic liver disease (ASH), has been reported in exosome-rich biochemical fractions from serum [67]. As components of exosomes, secreted miRNAs are intended to mediate inter-tissue communication, often following pathological challenges [68,69], and specifically to coordinate systemic responses between the primary tissue, which exports them and the cells at secondary sites [17]. Critically, there is already evidence for some species (notably miR-122, miR-34a and miR-200a) being similarly elevated with disease in both intra-hepatocyte tissue samples and serum/blood samples. This inter-tissue concordance encourages the view that liver-specific miRNA profiles of disease can be accurately assessed by interrogation of exosome fractions derived from more easily-obtained soluble biopsies.

We are unaware of any miRNA-based therapeutic candidate for NAFLD currently being tested by a clinical trial. Although many miRNAs represent attractive potential therapeutic targets for intervening in disease progression, the efficient delivery of an effector molecule remains a significant challenge for RNA interference-based approaches to treatment. As delivery of miRNA mimics or antagomirs may lead to rapid degradation of the naked molecules, it is likely that they will need to be incorporated into stable vehicles, such as nucleic acid lipid particles or lipid bilayers coated with polyethylene glycol or conjugated to cholesterol, in order to be taken up by liver [70,71]. However, the injection of miR-122 antagomir in the form of a “locked nucleic acid” (LNA)-modified oligonucleotide has been shown to suppress viremia in chronically hepatitis C viral-infected chimpanzees [72]. Indeed, an miR-122-based therapeutic approach has successfully undergone phase IIa human clinical trials for the treatment of hepatic viral infection [73], encouraging the view that the extension of miRNA-based approaches to NAFLD-related syndrome may be on the near horizon. 

**Table 1:** List of miRNAs involved in different biological processes of hepatic lipid homeostasis.

Biological Samples	miRNA(s)	Outcome	Target *	References
Plasma	miR-122	Inhibition of miR-122 reduced hepatic cholesterol and FA biosynthesis and elevated FA oxidation in humans	SREBP-1c, SREBP-2	[26,27,32]
Intracellular	miR-122, miR-451, miR-27a	Downregulated in the liver of high fat/fructose-fed rats	miR-451→NFκB	[34,74,75]
			miR-27a→PPARγ	
Plasma/Intracellular	miR-33a/b, miR-143, miR-92b	Inhibition of miR-33a/b increased HDL and lowers VLDL	SREBP-1, SREBP-2, AMPKα, IRS2, MSTN, FOXO1	[29,30,55]
		Overexpression of miR-33a/b increased HDL and reduces VLDL		
Plasma/Intracellular	miR-34a, miR-16, miR-21, miR-27b, miR-122, miR-192, miR-375, miR-19a/b, miR-146b, miR-181a, miR-200	Overexpressed in circulation of steatotic, NASH and T2DM humans/rats	miR-34a→p53	[32,33,61,62,76,77,78,79]
			miR-21→HMG-CoA	
			miR-27b→ PPARγ	
			miR-146b→IL-6, TNF-α	
Intracellular	miR-200a/b, miR-429	Upregulated in the liver of high-fat/fructose-fed rats		[34]
Intracellular	miR-29c	Downregulated in the liver of *ob*/*ob* mice on a lipogenic diet		[35]
Plasma	miR-192, miR-375, miR-122	Increased particularly in NASH, suppression of glucose-induced insulin secretion	miR-375→HMG-CoA	[32,62,80]
Plasma	miR-155, miR-122	Upregulated in rats with ASH	miR-155→LXRα	[67,81]

ASH: alcoholic steatohepatitis; FA(s): fatty acid(s); HMG-CoA: 3-hydroxy-3-methyl-glutaryl-CoA; NAFLD: non-alcoholic fatty liver; NASH: non-alcoholic steatohepatitis; SREBP: sterol regulatory element-binding protein; p53: Tumour protein p53; T2DM: type 2 diabetes mellitus; TG: triglyceride. * Genes are listed as human homologs.

## 4. Conclusions

While the precise molecular mechanisms underlying progression to NAFLD remain unclear, the emerging importance of miRNAs in the context of this disease suggests that they may represent valuable diagnostic tools. Although no miRNA-based trials are yet underway to address their utility as potential intervention strategies, a growing body of literature suggests that miRNA profiles reflective of disease progression and measured in readily-obtainable (least invasive) “liquid biopsies” offer considerable promise for assessing both risk and disease status along the NAFLD spectrum of pathologies. Critically, such tools would help to overcome current clinical limitations to the identification and accurate diagnostic assessment of “at risk” individuals, the numbers of which appear to be increasing as the epidemic of metabolic syndrome continues. 
